# Social beliefs and women’s role in sanitation decision making in Bihar, India: An exploratory mixed method study

**DOI:** 10.1371/journal.pone.0262643

**Published:** 2022-01-27

**Authors:** Sania Ashraf, Jinyi Kuang, Upasak Das, Alex Shpenev, Erik Thulin, Cristina Bicchieri

**Affiliations:** 1 Center for Social Norms and Behavioral Dynamics, University of Pennsylvania, Philadelphia, Pennsylvania, United States of America; 2 Global Development Institute, University of Manchester, Manchester, United Kingdom; 3 Center for Behavior and the Environment, Rare, Arlington, VA, United States of America; All India Institute of Medical Sciences Bhopal, INDIA

## Abstract

In low- and middle-income countries, poor autonomy prevents women from making financial decisions, which may impact their access to improved sanitation facilities. Inadequate access to improved sanitation disproportionately affects women’s and children’s health and wellbeing. Although socio-cultural factors are known contributors to gender inequity, social beliefs that potentially motivate or dissuade women from making sanitation-related household decisions are not well understood. These beliefs may vary across settlement types. To empower more women to make sanitation-related decisions, the relevant socio-cultural norms and underlying social beliefs need to be addressed. In this mixed methods study, we explored women’s role in sanitation-related decision making in three settlement types, urban slums, peri-urban, and rural communities in Bihar. Trained qualitative researchers conducted six focus group discussions with women of two age groups: 18–30 years old, and 45–65 years old to understand the norm-focused factors around women’s role in getting a toilet for their household. Using insights generated from these group discussions, we developed and conducted a theory-driven survey in 2528 randomly selected participants, to assess the social beliefs regarding women making toilet construction decisions in these communities. Overall, 45% of the respondents reported making joint decisions to build toilets that involved both men and women household members. More women exclusively led this decision-making process in peri-urban (26%) and rural areas (35%) compared to urban slums (12%). Social beliefs that men commonly led household decisions to build toilets were negatively associated with women’s participation in decision making in urban slums (adjusted prevalence ratio, aPR: 0.53, 95% CI: 0.42, 0.68). Qualitative insights highlighted normative expectations to take joint decisions with elders, especially in joint family settings. Surrounding norms that limited women’s physical mobility and access to peers undermined their confidence in making large financial decisions involved in toilet construction. Women were more likely to be involved in sanitation decisions in peri-urban and rural contexts. Women’s involvement in such decisions was perceived as widely acceptable. This highlights the opportunity to increase women’s participation in sanitation decision making, particularly in urban contexts. As more women get involved in decisions to build toilets, highlighting this norm may encourage gender-equitable engagement in sanitation-related decisions in low-resource settings.

## Introduction

Safe access to sanitation facilities, especially functional toilets, is fundamental for improved physical and mental health, wellbeing, and education outcomes [1,2]. Poor access to improved sanitation disproportionately affects women and girls over their life course. This includes a higher risk of violence, lack of privacy, increased psychosocial stress, potential health risks including risks to their reproductive health, and poor menstrual hygiene management [3,4]. Gender differences exist across the sanitation value chain, from limited access, frequency of use, child care needs, safe access to public toilets, to responsibilities of daily maintenance [5,6]. The need to increase gender equity in sanitation access is emphasized in the Sustainable Development Goals (SDG) 5 and 6 to ‘eradicate open defecation and ensure the availability and sustainable management of sanitation for all, with a specific focus on addressing the needs of women and girls and people in vulnerable situations.

Traditionally, women’s roles in household sanitation reflect caregiver roles including child feces disposal, water collection, and the responsibility to clean and maintain toilets [7–9]. Men are typically regarded as the primary wage-earners in the family and consequently make important financial decisions for the household [10]. Many of these decisions are influenced by gender norms which are culture- and context-specific and apply differently across life stages [11]. Studies have highlighted that these gender roles hinder women’s ability to negotiate favorable intra-household allocations of resources including those related to improved sanitation [4,7–9,12]. Substantial research suggests that existing family hierarchies which devalue female opinions, and societal factors that promote financial dependency constrain women’s participation in financial decisions [12,13]. A study from Nepal found that women who were older and had independent earnings had increased decision-making power on major household purchases [13]. Similar findings from Odisha, India, showed that women’s lack of negotiation power was related to low socio-economic status, and low confidence to make independent decisions [12]. A study in Kenya showed that women who made decisions for major household purchases were also more likely to live in a household with better sanitation [14]. Notably, improving women’s ability to make household decisions in low or lower-middle income countries was associated with positive health outcomes [15]. Specifically, studies have shown that if women were empowered to make household financial decisions, it led to improved child nutrition and growth [16], general well-being of women and girls [17,18], and overall hygiene indicators for the household [14].

### Social Norms Theory

Despite the known influence of sociocultural norms on women’s ability to engage in household decisions, specific social beliefs supporting the norms that potentially encourage or dissuade women from making sanitation-related decisions have not been systematically studied. To address this gap, we used the Social Norms Theory (SNT) framework [19] to understand which social beliefs drive women’s involvement in household sanitation decisions. The SNT framework identifies two types of social beliefs that influence behavior. The first is **empirical expectations**, or beliefs about what others in one’s reference group do. In this context, empirical expectations are measured as respondents’ belief of whether men decide to build a household toilet. The second type is **normative expectations,** or what respondents believe others in their reference network think one should or should not do. In this context, normative expectations are measured as respondents’ beliefs about whether other community members think women *should/should not* convince their families to build a toilet. In our study, we asked about women ‘convincing the family’ instead of leading the decision following formative research that suggested that even if women led the decision to build a toilet, it would be reported ultimately taken together with other family members in our study context. In addition to these two social beliefs, SNT considers that also **personal normative beliefs** may influence behavior, that is, one’s own belief about what should or should not be done. In this context, the personal normative belief is measured by asking respondents whether they think women should/should not participate in such household decisions.

Understanding the underlying social beliefs driving women’s participation in sanitation decisions is critical to inform appropriate gender-inclusive programs that can empower women, keeping in mind that targeting different social or personal beliefs require different intervention techniques [19]. These beliefs may also differ across communities or settlement types. If empirical expectations are associated with women’s participation in household decision making, highlighting these positive behaviors in their communities can promote women’s engagement by making it more acceptable. If normative beliefs, either normative expectations or personal normative beliefs, restrict women’s participation in household decision making, programs aimed to address the social stigma and associated social sanctions will be necessary. In this latter case, intervention strategies that engage norm enforcers like relevant family members to develop context-specific norm-focused activities would be necessary.

We recognized that gender norms can intersect with social factors differently across women’s life course and exert their influence across multiple domains of influence [11]. To take this into account, we used a framework influenced by Cislaghi et. al. to situate gender norms within wider levels of influence including overlapping domains (e.g. between the individual, their household, and their community) that are relevant when considering gender norms [20].

In this study, we aimed to understand 1) who leads the decision to build household toilets 2) what are the individual, household, and community-level factors that may influence women’s sanitation decision making 3) which social and personal beliefs are associated with women’s participation in financial decision-making in our study context 4) whether these beliefs differ by settlement types across urban, peri-urban, and rural communities.

## Methods

### Study design

**Fig 1:**
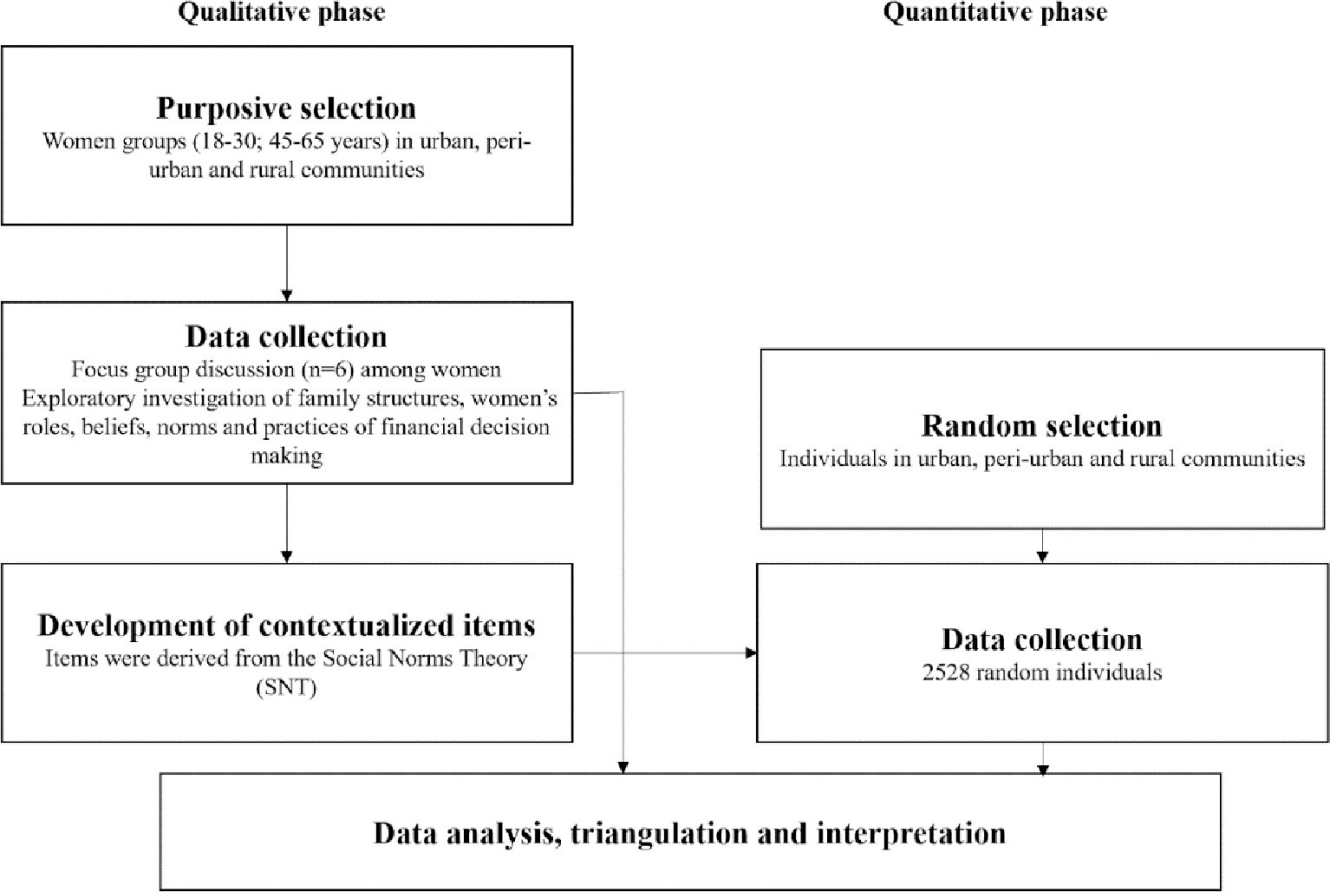
Exploratory mixed method study design, Bihar, 2018.

We used a sequential exploratory mixed methods design to address our research aims. We first conducted exploratory qualitative research, followed by a quantitative research phase in three settlement types: urban slums, peri-urban and rural communities in Bihar (Fig 1). In the qualitative phase, we used focus group discussions to understand family structures, household roles for younger and older women in rural, peri-urban and urban communities in Bihar, and the descriptions of the financial decisions they make. We also elicited from the female participants their social beliefs and the perceived sanctions from household and community members if they led toilet construction decisions. Next, we used the insights to refine the language and contextual nuance of the survey items used in the subsequent quantitative phase. We integrated the data during interpretation to triangulate our findings.

### Study site and population

This study was conducted in Bihar, which is an eastern state of India. This state is known to lag behind in terms of economic growth, health outcomes, education attainment, and gender equality compared to the rest of the country [21,22]. In 2016, only an estimated 25.2% of households used improved sanitation facilities in Bihar [23].

### Qualitative research methods and analysis

#### Sample selection

We purposely selected one community in three settlement contexts, rural (Gram Panchayat), peri-urban (Town Panchayat), and slums in urban communities (Municipal Corporation) to explore social beliefs across different levels of urbanization in the Patna district of Bihar. The research team contacted local authorities to seek permission to recruit eligible participants for focus group discussions (FGD). Field assistants went house to house to seek permission from relevant household members and recruit eligible women between the age of 18–30 or 45–65 years old for separate focus group discussions. We separated these two age groups to allow younger women to voice their opinions freely in front of their elders, given that the respondents might know each other within the same locality and follow social norms based on family- and age-based hierarchies. For younger women, the participants were: unmarried women living with their parents, newly married, or a new mother living with their in-laws. For the older age group, we included mothers-in-law and daughters-in-law with children. Members from both nuclear and joint families, defined as extended families of two or more generations living as a single household, were included in these FGDs. Respondents were screened to ensure they were available for up to an hour and willing to participate in the discussion.

#### Data collection

For each of the settlement types, we conducted two FGD with participants women of 18–30 years old and women of 45–65 years old, respectively (n = 6). We developed a theory-driven, semi-structured discussion guide that we contextualized based on relevant literature. The discussion guides were designed to allow exploration of prevalent practices, norms, and beliefs surrounding sanitation and gender dynamics in the communities (S1 for FGD guides). We examined the following topics: 1) Prevalence of open defecation and the women’s experience in the community (where people go, personal experiences, reasons for open defecation), 2) issues related to sanitation facilities (i.e., access, use, ownership, cleaning, community toilet use), 3) decision making at the household level and associated beliefs (i.e., who decides, what type of decisions are made by women vs. men, experiences or examples of decision making related to toilet construction); 4) perceived opinions about women taking independent decisions to build a toilet (reactions from the community, from within the household).

A team of four qualitative researchers with experience working in Bihar reviewed and provided feedback over a 5-day training period prior to the field activities. Experienced moderators used the focus group guides to conduct the discussions in the local language, Hindi. These focus group discussions were conducted across two weeks in March 2018. These discussions took 60–70 minutes and were conducted in a secured private room in a school or a community center, selected in collaboration with local community members. All participants provided oral consent at the start of the session. All sessions had a designated note-taker and were audio-recorded. The audio recordings and field notes were transcribed and translated into English by a third party for analysis. Oral consent was obtained from all participants prior to the focused group discussion. This study was approved by the Institutional Review Board at the University of Pennsylvania (Protocol #: 827239) and by the Social Research Institute (IRB registration number: IORG0009562) in India, which served as the local IRB for our study.

#### Analysis

The data were transcribed verbatim and translated into English. Two researchers independently conducted thematic context analysis on the qualitative transcripts. We used theory-driven codes to reflect psychosocial, contextual, and norms-focused categories derived using Bicchieri’s social norms theory. We also added new codes that emerged from the data we gathered. Code refinement was conducted following consultation with research investigators. We developed a framework guided by SNT and Cislaghi et al. (19,20) to organize and summarize the data by several factors across levels of influence. Relevant quotes illustrating the findings were identified. During the interpretation phase, we integrated the findings from quantitative analyses and used them to triangulate findings towards study aims.

### Quantitative research methods and analysis

#### Sample selection

**Fig 2:**
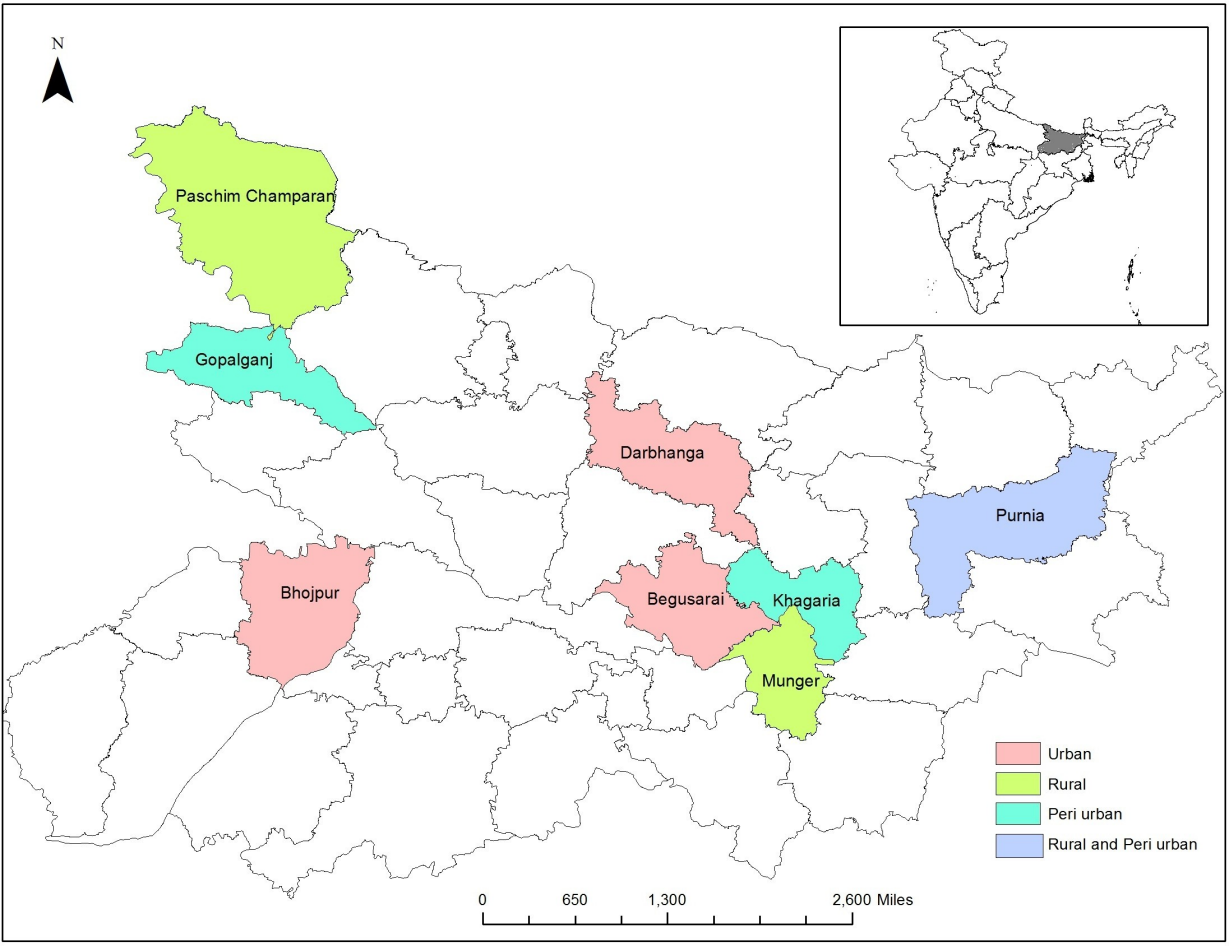
Study sites included in this study, Bihar, 2018.

We conducted our study in 8 purposively selected districts, geographically spread out across Bihar to provide a range of socio-cultural environments with respect to variations in language and general societal practices. For the rural sample, we selected three districts (Purnia, Munger and Paschim Champaran) and used the complete list to select two Gram Panchayats. The first community was randomly selected and the second one was systematically selected by matching key socio-economic characteristics that include the population size, proportion of agricultural laborers, illiterate individuals, and households with toilets along with the proportion of Scheduled Caste and Scheduled Tribe groups, based on the Census of India, 2011. We followed a similar selection strategy for the peri urban sample, choosing Nagar Panchayats from 3 districts (Purnia, Khagaria and Gopalganj) and Municipal Corporations from 3 districts (Darbhanga, Begusarai and Arra) for the urban slum sample (Fig 2). We did not include households residing in non-notified slums.

In summary, the survey was administered in 30 randomly selected sampling units. The sample was drawn from three settlement types, including six rural communities (Gram Panchayats), eighteen semi-urban communities (census wards from six Nagar Panchayats), and six urban communities (registered slums).We generated a complete listing of respondents in dwelling units or households and randomly selected eligible individuals between 16–65 years in the selected areas. These sampling units were considered as proxies from communities, Gram Panchayat for rural, registered slums for urban and census wards in Town Panchayats for peri-urban areas.

#### Data collection

Informed by the qualitative findings, we revised norms-focused survey items to assess empirical expectations, personal normative beliefs, and normative expectations regarding women inducing their families to build a toilet. We tested the survey questions among men and women from similar communities to assess comprehension and made relevant revisions. A group of bilingual researchers translated the survey items to the local language (Hindi) and back-translated to English. Any inconsistencies were addressed to ensure the validity of the items. All field workers received training to ensure a standardized survey collection procedure. Field workers then surveyed 2528 randomly selected individuals from 30 communities in Bihar between April-June 2018. The survey was administered with Computer Assisted Personal Interviewing (CAPI) on hand-held tablets. Consent forms were read out to all respondents and oral consent was obtained prior to starting the surveys and focus group discussions. A copy of the consent form was provided to the respondent.

#### Measurements

*Demographic characteristics*. We collected data regarding respondents’ gender, age, education attainment, self-identified caste and religious group, and current employment status. We collected information on possession of assets in line with what is collected in the National Family Health Survey (NFHS) conducted by the Ministry of Health and Family Welfare of the Government of India. However, given the distribution of possession across households, we found discriminatory variation in the possession of color television, internet, motorized two-wheeler, and refrigerator and not for other assets. For instance, in our study sample, majority (96.7%) did not own a computer or laptop. Similarly, possession of a car (0.8%) and air conditioners (1.3%) were low. For the four selected assets for socio-economic status (SES), we noted a considerable level of ownership i.e. color television (44%), motorized two-wheeler (22%), refrigerator (11%), and internet (27%). We used three SES categories: low for households who own none of these items, medium for those own at least one item, and high for those who own at least two of these items.

*Household sanitation decision*. We first asked the respondent if they owned a toilet and what kind of toilet they usually used. Among those who reported owning a toilet, we asked “who in your household got the family to build a toilet?”. The answer options include male, female, mutual decisions. We coded women participating in household sanitation decisions as a binary variable (1 = female/mutual, 0 = male). The framing of this question was guided by qualitative work preceding the surveys to capture women’s decision making for household toilet construction.

*Social beliefs of sanitation decision*. To measure empirical expectations (i.e., beliefs about what other people do), we asked all respondents “Out of ten households in your community who have toilets, in how many do you think a male household member got the family to build a toilet?” We measure respondents’ normative expectations (i.e., what an individual believes other people think one should do) with a similar style question: “Out of ten members of your community, how many do you think believe that it is wrong for women to get her family to build a toilet?”. The responses ranged from 0 to 10, where 0 represented the respondent thinks no households/community members did/believed so and 10 represents the respondent thinks all households/community members did/believed so. We linearly transformed (divided by 10) these two scales to a 0 to 1 scale to represent perceived empirical/normative prevalence, respectively. Finally, we measured respondents’ personal normative beliefs (i.e., what an individual personally thinks one should do) by asking a balanced question to reduce social desirability bias “Society may think it is right or wrong for a woman to get her family to build a toilet. Do you personally think it is right, neither right nor wrong, or wrong for a woman to get her family to build a toilet?”.

#### Analysis

To assess the prevalence of women making household decisions and the surrounding social beliefs, we calculated descriptive statistics of sample respondents by settlement types and gender. To examine the difference in social beliefs across gender and settlement types, we used Pearson chi-square tests for discrete outcome variables and Kruskal-Wallis test for continuous variables. We also used Mann-Whitney tests for pairwise comparisons of settlement types with Bonferroni correction as a post-hoc measure. To assess factors associated with household sanitation decisions and social beliefs we used multivariable regression models to control for potential confounders such as respondents’ gender, age, education attainment, socioeconomic status, and socio-religion group. In addition, we accounted for settlement type differences through urban, peri-urban or rural sector dummies and the corresponding community fixed effects (slums for urban; census wards for peri-urban and GPs for rural areas). With these extensive set of controls, in the final model, district level controls dropped out due to multicollinearity and only community level cluster adjustments were retained. We used the robust variance estimates to adjust for clustering at the community level. We normalized responses to the empirical expectation item (range 0–1) in each settlement type to allow us to compare them to each other. All analyses were conducted using Stata v. 14 (StataCorp. LP) and R v.3.6 (R Core team 2020).

## Results

### Qualitative

A total of 51 women participated in the focus group discussions (Table 1). Around 55% of the participants were from joint families, i.e. extended families of two or more generations living as a single household. Participants with a private toilet believed many households in their communities had toilets or had applied to get new toilets. In villages and peri-urban communities, the community layouts were segregated considerably by caste.

**Table 1:** Socio-demographic characteristics of participants in focus group discussions, Bihar, 2018.

No.	Focus group discussion	Settlement type	Participants (n)	Approximate age in years (mean, sd)	Household size (mean)	Household toilet ownership (%)^1^	Composition
1	Younger group (18–30 years)	Rural	8	24 (5)	7	50%	Joint families (n = 6), Nuclear families (n = 2), Had children (n = 2)
2	Younger group	Peri-urban	9	23 (3)	7	44%	Joint families (n = 7), Nuclear families (n = 2), Had children (n = 4)
3	Younger group	Urban	8	26 (4)	5	88%	Joint families (n = 6), Nuclear families (n = 5), Had children (n = 2)
4	Older group^2^ (45–60 years)	Rural	10	46 (5)	6	60%	Joint families (n = 3)
5	Older group	Peri-urban	8	52 (5)	7	63%	Joint families (n = 3)
6	Older group	Urban	8	42 (4)	5	75%	7 nuclear families

^1^Collected during screening for focus group discussion recruitment; Caste or tribe was not asked due to avoid any social sensitivity during the group discussion.

^2^ All women in older age groups in our study reported living with their grown children’s families.

We noted variations in women’s experiences of engaging in the decision-making process to build a toilet, depending on individual and interpersonal factors such as women’s age and social role in the family (e.g. daughter, daughter- in-laws, mothers-in-law). These experiences were further influenced by societal and community-level factors depending on the settlement type and perceptions of common practices in their community unities. We summarized our findings using an ecological framework consisting of four levels (institutional, community, household/interpersonal, and individual) with contextual, psychological, and material/technological domains guided by SNT and Cislaghi et al. [19,20] (Table 2). This qualitative summary aimed to describe the various channels through which norms exert their influence on women’s ability to make sanitation decisions. Since we conducted a small number of FDGs, we describe the presence of these factors but are limited in extrapolating the extent of their influence.

**Table 2:** Summary of qualitative findings from focus group discussions, Bihar, India 2018.

	*Levels*	*Contextual factors*	*Psychosocial factors*	*Technology/Material factors*
	***Institutional***	Settlement type Swachh Bharat Mission [e.g. mass media promotion of building toilets for families and women]	Perceived cost of toilets	Access to sanitation markets
***Social***	***Community***	New toilets were built in one’s community. Lack of access to community Restricted physical mobility^†^	Descriptive norms [beliefs about other women’s role in getting a household toilet] *^†^	
	***Interpersonal/*** ***household***	Joint or nuclear families Intra-household social hierarchy Existing caregiving roles and responsibility Husbands/elders were primary financial decision makers Financial stress	(Need for) approval from elders/spouse for major decisions (Lack of) injunctive norms/normative expectations [Beliefs about whether others approve of women’s involvement in household decisions to construct a toilet] * Social support^††^	Lack of space
	***Individual***	Age Education Employment status	Self-efficacy to handle money Experience of making large financial decisions (Lack of) Physical mobility^†^ Exposure to role models^†^ Personal normative beliefs [beliefs men should make large financial decisions] * Perceived benefits Perceived bargaining power^††^ Competing priorities	Factual beliefs about how much a toilet/mason services cost* Knowledge about the process of constructing a toilet

*Derived from Bicchieri’s Social Norms Theory.

^†^Can be conceptualized as an intersection of the community and individual level factors (*Cislaghi et*. *al*.*)*, where the women have low social exposure to role models.

^††^Can be conceptualized as an intersection of the individual and household level (*Cislaghi et*. *al*.*)*, where women might be able to influence toilet construction during weddings or through children.

In general, rural participants emphasized the presence of stronger gender norms that restricted women’s autonomy and mobility outside the house if they were newly married daughters in laws. Women from peri-urban or urban areas had more opportunities to work, had access to social networks, and were more aware of the process of building a toilet. When exploring who led the decision for toilet construction, spatial and financial barriers were highlighted as factors that complicated the role of women to convince their families to construct a toilet. Norms and social beliefs such as these represented intersecting factors between the individual, household, and community levels (Table 2).

At the institutional level, Swachh Bharat Mission increased awareness of toilet promotion and construction through increased access to sanitation markets. The subsidies impacted the perceived cost and ease of building a toilet. At the social level, we categorized our findings at community and household- levels to illustrate the emerging social beliefs related to women’s participation in toilet construction. Descriptive norms of perceived prevalence of households building a toilet and women’s role in deciding to build them were important factors at the intersection of individual and the community levels.

Most women’s influence involved motivating the need for a toilet to encourage family members to agree to build one. They also engaged directly or through family members in the process of applying to receive a subsidy and hiring a mason to complete the construction. In the next section, we detail household/interpersonal factors that emerged from our qualitative data.

#### Family structures

Many respondents from rural and peri-urban communities shared that they lived in joint families, usually consisting of grown-up children, parents, and their spouses. This meant sharing meals, living spaces, and sharing financial responsibilities. The qualitative data suggested that the role of family elders was important, where their roles included giving permission or being consulted for marriages, ceremonial rituals, large purchases, or construction. There was a shared consensus that the support of family members was necessary, regardless of gender, in any big financial purchase, such as building a toilet. We found evidence that families who lived in joint households relied on the permission or approval of their elders to proceed with major decisions, like building a toilet.

Women in both urban and rural areas reported joint decisions to build a toilet. The ability to convince household members was more pronounced in women who had some income of their own or those who lived in nuclear families. Since building a toilet included construction associated with the household structure, involvement of other family members was expected and required.

*“Usually*, *it’s a joint decision*. *Even if a woman has the money to build a toilet*, *they must ask the household members*.*”—*35-year-old woman in peri-urban Bihar

#### Gendered roles of women

Women performed traditional roles where daughters-in-law took care of household chores with guidance from the mothers-in-law. Younger women, especially those unmarried or students in urban areas, described going to school as well as participating in chores. In rural areas, we found evidence that women were traditionally in charge of smaller expenses like grocery, shopping, or maintenance costs, while men oversaw large purchases like electronics or construction.

In rural areas, there was evidence of limited mobility outside the house, particularly prominent for new daughters-in-laws. They needed to be accompanied by a family member to go out to defecate, or even do grocery shopping nearby. While the demand to build a private toilet in a household was often driven by the female members, many lacked the self-confidence or perceived self-efficacy to convince relevant family members to implement the construction of the toilet (dealing with labor, materials, and funds from the government). These capabilities were influenced by education, income, or experience handling money. The ability to implement was driven by exposure to markets and related sanitation information, something that may be influenced by their environment.

One participant said: “*Now we are not capable of constructing the toilet as we are dependent on our husbands*. *We lack money*. *We also don’t have any land which we can sell and use the money for constructing a toilet* …*and that is why we are still waiting*.”—45-year-old woman in rural Bihar

There were qualitative indications that women with higher autonomy were those with a job or a source of income. We found evidence that some women were able to access financial services and get support from their family members to complete the toilet construction.

*“I made my own latrine by taking a loan—my son and my father brought the materials and brought the mason—I stood there while they made it”—*38-year-old woman in peri urban Bihar

#### Social hierarchies in families

In joint family settings, the seniority of the female members (mother-in-law vs. daughter-in-law) played a role in their ability to make this decision.

*“Only older women can convince the men then that’s good—the bahu [daughter in law] doesn’t have bargaining power right away”*—a 65-year-old woman from peri-urban Bihar

Some women without education perceived their primary role in the decision-making process to be limited to convincing their spouses or their male family members to build a toilet.

*“There are no decisions that only women can make*. *[they] must go through the husband or the son*.*”—*a 65-year-old woman from peri-urban Bihar

#### Women’s bargaining power

We found that there was a perceived opportunity for young women to demand toilets when deciding whether to marry into a family. The participants knew or heard of this opportunity from their social networks. This appeared to be a strong motivator to convince families to build a toilet, one that was driven by women.

*“Person 1*: *Nowadays everyone comes to check the toilets in the house before marrying their daughter in that house*. *If that house has the toilet then it is ok but if that house does not have the toilet then there is a problem*.*” — 60-year-old woman in rural Bihar*

*“Person 2*: *If that house does not have the toilet then people look for some other house with toilets to marry their daughter*.*”—55-year-old woman in rural Bihar*

#### Perceived social support to make sanitation decisions

Women told anecdotes about their own families or others in their communities who convinced their families to build a toilet. In addition, when women had to convince their family, they recalled other family members or community leaders who helped them in these decisions.

*“Person 1*: *If we want to [*…*build a toilet*‥*] we will*. *If the [family] doesn’t support us we can find the support to do this*. *Other families have done it*.*”* —*26-year-old*

“Person 2: *The husband and in-laws didn’t support her*. *My sister helped her*, *went with her to discuss with them and convinced them*.”—*30-year-old*

*“Person 3*: *If I want something*, *I have to convince my husband*. *My husband then discusses it with his parents*. *If the husband or the in-laws do not agree I know someone whose family member came and convinced them*.*” 28-year-old*, *urban town in Bihar*

We also found evidence that children were a source of motivation for women to influence their families to build a toilet. Many women reported their young and teenage children were asking the parents to build toilets for their use.

Based on the qualitative insight that many women were primarily involved in convincing their families, or the decision maker, to build a toilet, we framed the outcome of interest in the quantitative assessment of social beliefs as “a woman convincing her family to build a toilet”. The translated versions were piloted with similar respondents to ensure comprehension with respect to the decision-making role regarding toilet construction.

### Quantitative

Field workers surveyed 2533 respondents. After excluding those with missing answers (n = 5), we included 2528 respondents (women = 52%) from urban slums (n = 832), peri-urban (n = 867), and rural (n = 829) in our analysis. The average age of the included respondents was 35 (SD = 14). Among them, 75% are Hindu, 54% have received some education. Most men worked in agricultural or industrial jobs (n = 530, 44%) while most women were homemakers or retired (n = 1060, 81%). Most households owned the house (95%), had electricity (93%), and used tube wells or boreholes (86%) as the main source of drinking water. Less than half of the households had a separate room that was used as a kitchen (31%) and owned a private toilet (47%). A few households (12%) reported sharing the ownership of a toilet with other households. Among those who own a toilet, most of its toilets were functioning (98%), and about half were constructed more than 3 years ago (53%). The demographic characteristics of the respondents and household characteristics are described in Table 3.

**Table 3:** Characteristics of the study population in urban slum, peri-urban and urban areas, Bihar, 2018.

Variables N (%)	Total (N = 2528)	Urban Slum (N = 832)	Peri-urban (N = 867)	Rural (N = 829)
**Age** mean (sd)	35 (13)	34 (14)	35 (14)	36 (14)
**Female**	1311(52)	421(51)	432 (50)	458 (55)
**Education**				
No formal education	1159 (46)	375 (45)	332 (38)	452 (55)
Primary (1–5 years)	626 (25)	195 (23)	222 (26)	209 (25)
Secondary (6–10)	298 (12)	82 (9.9)	129 (15)	87 (11)
High school (11–12)	271 (11)	103 (12)	111 (13)	57 (6.9)
College or above (12+)	174 (7)	77 (9.3)	73 (8.4)	24 (2.9)
**Occupation**				
Salaried workers/business owners	464 (18)	191 (23)	169 (20)	104 (13)
Agricultural/skilled workers	626 (25)	170 (20)	199 (23)	257 (31)
Student	285 (11)	103 (12)	104 (12)	78 (9.4)
Homemakers/Pensioners/Retired	1153 (46)	368 (44)	395 (46)	390 (47)
**Socio-religious group**				
Hindu upper caste	147 (5.8)	47 (5.6)	53 (6.1)	47 (5.7)
Hindu scheduled caste	627 (25)	414 (50)	102 (12)	111 (13)
Hindu others	1090 (43)	213 (26)	528 (61)	349 (42)
Muslim and other religions	664 (26)	158 (19)	184 (21)	322 (39)
**Socioeconomic status**				
Low	1254 (50)	348 (42)	383 (44)	523 (63)
Medium	734 (29)	303 (36)	260 (30)	171 (21)
High	540 (21)	181 (22)	224 (26)	135 (16)
**Household size** mean(sd)	8.5 (2.8)	8.6 (2.9)	8.3 (2.8)	8.6 (2.8)
**Owns the house**	2395 (95)	782 (94)	818 (94)	795 (96)
**Drinking water source**				
Public tap standpipe	205 (8.1)	105 (13)	37 (4.3)	63 (7.6)
Tube well or borehole	2169 (86)	705 (85)	752 (87)	712 (86)
Other	124 (4.9)	28 (2.2)	67 (7.8)	39 (4.7)
**Toilet ownership**				
No latrine	1043 (41)	314 (38)	259 (30)	470 (57)
Sole owner	1181 (47)	363 (44)	510 (59)	308 (37)
Shared with other households	304 (12)	155 (19)	98 (11)	51 (6.2)

#### Household sanitation decision making

**Fig 3:**
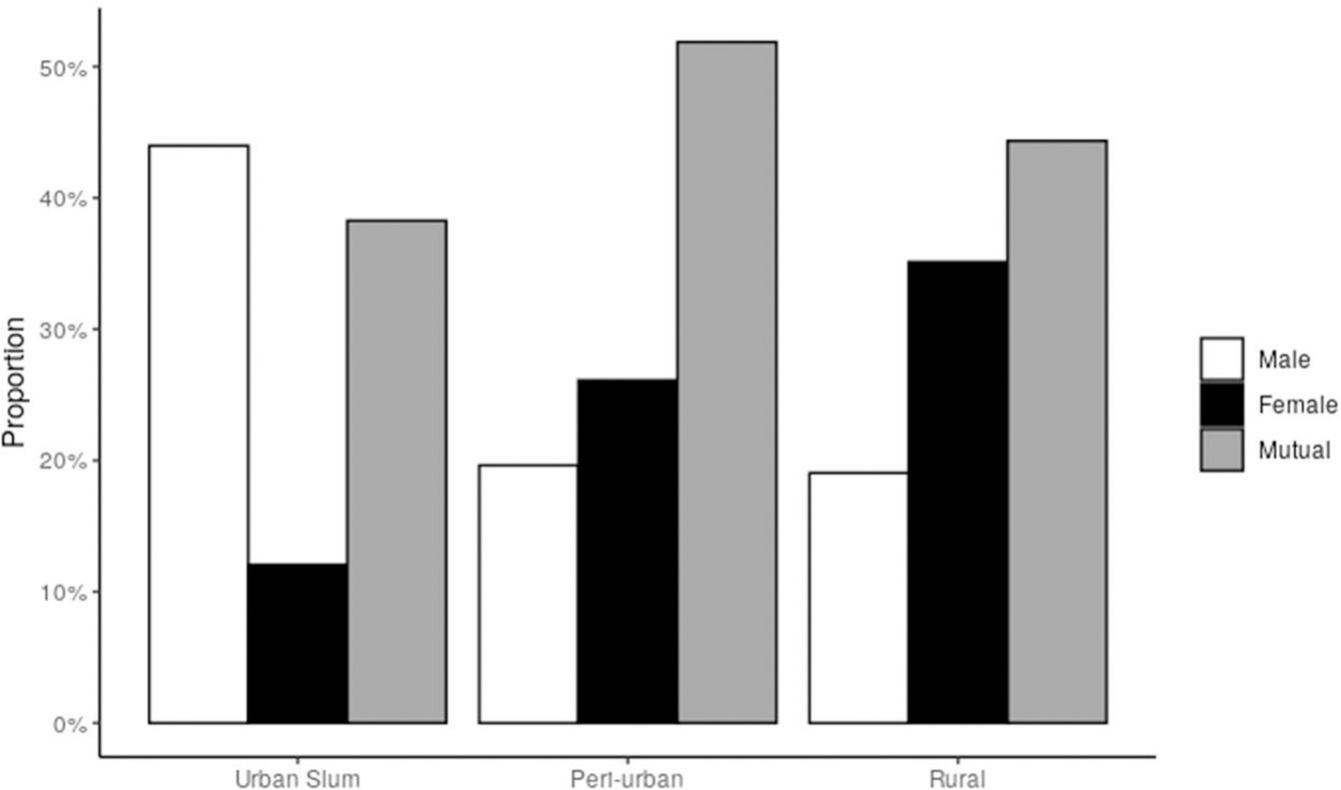
Decision to get a household toilet among toilet owners (N = 1485) by gender and settlement types, Bihar, 2018.

Among 1395 respondents who reported owning a toilet, a considerable proportion (45%) reported joint decisions to build a toilet (urban slum: 38%, peri-urban: 52%, rural:44%). In urban slums (n = 473), exclusive decisions by men were more common (44% men vs 12% women), whereas in peri-urban (n = 586) and rural areas (n = 336), more women were exclusive deciders (peri-urban: 19% men vs 26% women; rural areas: 19% vs 35%) (Fig 3).

#### Social beliefs

**Fig 4:**
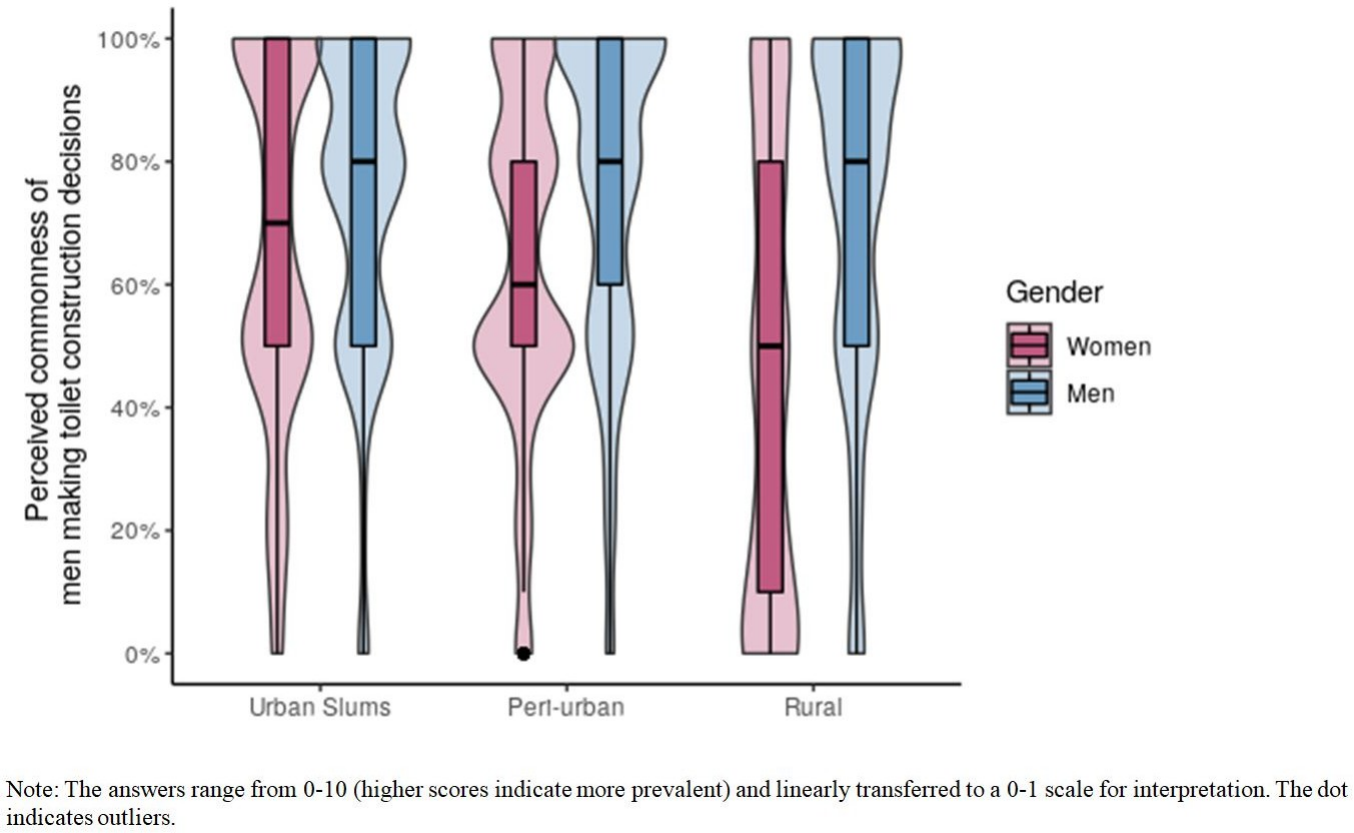
Social beliefs of household toilet decision making across different settlement types, Bihar, 2018.

*Empirical expectations (Beliefs of what others do in one’s community)*. Of 2528 respondents, a majority (66%) held the empirical expectation that men led the sanitation decisions in their communities. This social belief differed significantly across settlement type (Fig 4). Respondents from urban slums (mean = 0.71, sd = 0.28), and peri-urban areas (mean = 0.70, sd = 0.28) perceived that it was more common for men to lead sanitation decisions (p<0.001) compared to those from rural areas (mean = 0.57, sd = 0.36). This difference was not significant between peri-urban communities and urban slums (p = 0.46).

Compared to female respondents, males perceived that it was more common for other men to get the family to build a toilet (urban slum: 75% vs 68%, p = 0.01; peri-urban: 76% vs 64%, p<0.001; rural:72% vs 46%, p<0.001). In particular, men’s (mean) beliefs were consistent across settlement types while women’s beliefs varied considerably. Women in urban slums (68%) and peri-urban (64%) held similar empirical expectations. However, in rural areas, on average women believed less than half the decisions were led by men (46%) with a wide distribution around the mean.

**Fig 5:**
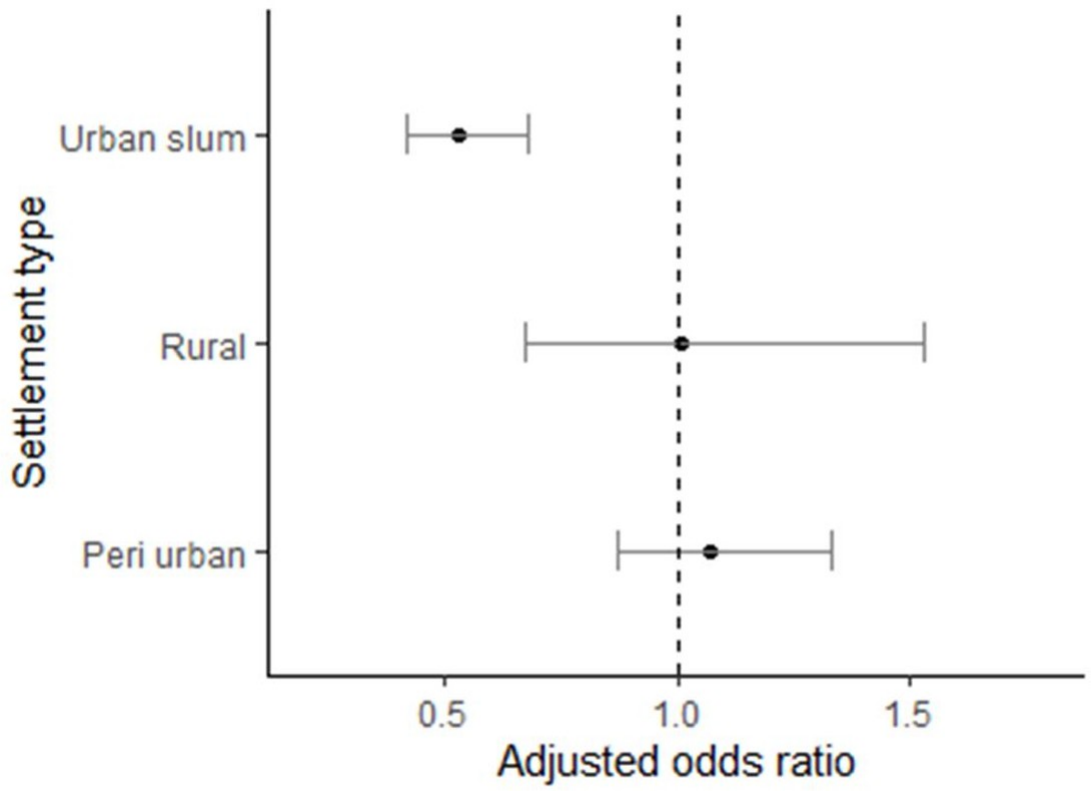
Multivariable regression assessing the influence of empirical expectations on female participation in toilet construction decisions with robust clustered standard error and community level fixed effects.

In multivariable regression models, we found that higher empirical expectations of men leading sanitation decisions were negatively associated with women leading similar decisions to get a household toilet (OR = 0.78, 95% CI: 0.61–0.99, p<0.05), controlling for respondents’ gender, education, caste, socio-economic status, household size, and community level fixed effect (Fig 5). In stratified analysis by settlement types, we found that social beliefs that men commonly led household decisions to build toilets were negatively associated with women’s participation in decision making in urban slums (adjusted prevalence ratio, aPR: 0.53, 95% CI: 0.42, 0.68) (Fig 5). This relationship did not reach significance in either peri-urban areas (aOR = 1.06, 95%CI: 0.86, 1.31) or rural areas (aOR = 1.05, 95% CI: 0.71, 1.55).

*Normative expectation (beliefs about what other people think one should do)*. We found evidence across multiple items that indicated that it was acceptable for women to make decisions to build a toilet for their household (Table 4). Out of 2528 respondents, only 19 (0.8%) respondents said they believe it was wrong for a woman to get her family to build a toilet. 74% said they believe no one in their community believed it is wrong for a woman to make sanitation decisions (72% among women vs 76% among men, p-value = 0.03). Only a small proportion (9%) believed that more than half of their community members believe it is wrong for women to make such decisions. These results suggest there is no evidence of normative constraints on women making decisions about toilet construction.

**Table 4:** Social beliefs about toilet construction of study population in urban slums, peri-urban, and rural areas, Bihar, 2018.

	Total (N = 2528)	Women (N = 1311)	Men (N = 1217)	Urban Slum (N = 832)	Peri-urban (N = 867)	Rural (N = 829)
**Empirical Expectation** ^1^						
Mean (sd)	0.66 (0.32)	0.59 (0.33)	0.74 (0.27)	0.714 (0.28)	0.698 (0.28)	0.57 (0.36)
**Personal normative belief** ^2^ **n (%)**						
Right	2380 (94%)	1232 (94%)	1148 (94%)	781 (94%)	835 (96%)	764 (92%)
Neither right nor wrong	129 (5.1%)	74 (5.6%)	55 (4.5%)	45 (5.4%)	28 (3.2%)	56 (6.8%)
Wrong	19 (0.8%)	5 (0.4%)	14 (1.2%)	6 (0.7%)	4 (0.5%)	9 (1.1%)
**Normative expectation** ^3^						
Mean (sd)	0.07 (0.28)	0.06 (0.26)	0.07 (0.29)	0.07 (0.28)	0.04 (0.22)	0.089 (0.32)

Note: ^**1**^Empirical expectation is the expectation that men in other households got the family to build a toilet;

^2^Personal normative belief is a belief about whether it is wrong for a woman to get the family to build a toilet;

^3^Normative expectation is the expectation that other people believe it is wrong for a woman to get the family to build a toilet.

## Discussion

Changing social beliefs about sanitation decisions may motivate increased participation of women to improve household sanitation conditions. In the setting of a national sanitation program (SBM) promoting toilet construction and use, we found that it was common for women to report joint decision making for toilet construction with male family members in Bihar. A higher proportion of rural and peri-urban women reported exclusively led toilet construction decisions compared to women in urban slums. This may be due to considerable social, spatial, and institutional barriers to toilet construction in urban slums such as negotiations with landlords, restrictive permissions, or lack of space to construct [24].

Our findings suggest that men consistently perceived that it is common for men to led sanitation decisions, while women’s social beliefs about who made such decisions varied. This divergence in expectations may be due to varying levels of exposure to this information across settlement types. In urban slums, correct social beliefs that men commonly led toilet construction decisions in their community were associated with fewer women making exclusive decisions in urban slums. This suggests important avenues for norm-focused behavior change strategies. The increased participation of women is recent and may be a consequence of the then ongoing national sanitation program (SBM), which leveraged social marketing and gender-focused messaging to encourage toilet construction for women.

In our study, we found qualitative evidence that highlighted a reinforced bargaining power of women during weddings, where the groom’s family felt social pressure to build a toilet if they did not own one. In Haryana, a study highlighted similar facilitators of toilet construction [25]. Indeed, the relatively high proportion of joint or women-led decision making may not be well known in these communities and could be an important psychosocial facilitator to enhance women’s self-efficacy in improving their household’s sanitation conditions. Broadcasting social information and highlighting similar women’s involvement in the decision-making process (positive descriptive norms), might empower more women to engage in decision making mechanisms traditionally reserved for men. Using positive case studies of female role models from one’s own community, who have led toilet construction, might motivate others to do the same. These conversations situated within one’s social networks can facilitate effective shifts in norms [20]. Community-wide public commitment activities that praise women decision makers or share testimonials from families who appreciate this role of female family members can be considered in behavior change strategies. Use of social media or text messages can also be explored to reach women who have limited mobility outside the house.

We notably found that women’s participation particular to toilet building decisions was viewed as acceptable and few expected that others would disapprove of it. Previous studies showed that involving women in household-level decisions can lead to better social and gender equity outcomes and have positive impacts on health and wellbeing [15]. In the absence of restrictive normative expectations, one key implication is that programs and practitioners who want to encourage women to participate in sanitation decision mechanisms will not need to shift normative expectations or beliefs around women’s participation.

That said, there are other considerations to address given that women’s role in decision making is qualitatively impacted by their social roles in the family, age, income, and their communities as reflected by where they lived (urban vs. rural). These factors are consistent with results from other sanitation-focused studies in Orissa [12] and Kenya [14]. For example, older or socially senior women in joint families or female household heads may have increased participation. In this study, we did not specifically capture the age, employment status or social position of the decision maker within the family limiting our ability to quantitatively assess the influence of these factors. In Bihar, it is common for women to live in joint patrilocal families, especially in rural areas. We found qualitative evidence that this may manifest in social barriers that reduce their access to peers through whom they could gather social information. These barriers were described in other studies that found women face mobility constraints outside the house that minimizing opportunities to learn from more progressive peers [26]. Studies from Uttar Pradesh have highlighted similar social norms hindering physical mobility that limited the effectiveness of interventions aimed to increase women’s autonomy [27]. The downstream consequences of limited household-level bargaining power are negative insofar as they may impact health outcomes in children and individual level choices such as contraceptive use [27–29]. Intra-family dynamics may strengthen norms that influence women’s ability to engage in household decision making. In such cases, context specific approaches are required to address these household level barriers to increase women’s empowerment.

We acknowledge that social beliefs operate within a complex framework of individual, household, and societal domains, and therefore only partially impact women’s participation in household sanitation decision making. Further research needs to take a holistic perspective that integrates a wide array of intersecting factors including norm-related components and underlying social beliefs in women empowerment and sanitation programs. Programs that address gender and social norms need to be mindful that these norms interact with other context-specific in resource-poor settings.

Our study has limitations. First, we only captured the male perspective about women’s participation through surveys, and not in qualitative discussions. Qualitative insights from the male perspective about the related norms would supplement our findings. Second, cross-sectional data only allow us to comment on the correlational nature of social beliefs and not temporal changes in beliefs. Next, we did not ask respondents directly whether they made the decision to build a toilet. Reported decisions from other household members may be subject to bias, especially if it was believed that an older female may have been involved. Collecting information on the social standing of the household member is important to better understand social dynamics. In exploratory analyses, adjusting for the age of the respondent in the multivariable model did not meaningfully change our effect estimates. In addition, the option “mutual” for decision making did not allow us to closely examine the impact of women’s opinion matters in the joint decision process. In this condition, women could take either the leading role or a supporting role during the decision-making process. We also acknowledge that self-reporting of exclusive decision making may be subject to bias, especially in patriarchal societies where reporting joint decisions may be perceived to be more acceptable. Social desirability bias in answering such gender-sensitive questions may have deflated the proportion of exclusive female-led decisions in our study. Moreover, respondents may be hesitant to acknowledge normative constraints during in-person surveys. In qualitative investigations, we divided FGD participants by age groups to limit these biases and cross-checked our quantitative and qualitative data. Finally, our study is only representative of our sample areas but not of the entire state. The questions about who convinced their families to build a toilet were only asked to households with a toilet. Inferences made from these households might not account for important contextual factors across settlement types. In addition, we selected one settlement type from each district. Although district level controls were not significant during our analyses, our findings may be subject to bias where the impact of inter-district differences guided by long standing cultural norms are not adequately addressed. Nonetheless, these insights generated through this paper can inform context-specific, inclusive intervention approaches that explores and leverages social beliefs to promote women’s empowerment.

## Conclusions

Women were substantially involved in exclusive sanitation decisions in peri-urban and rural contexts in Bihar. This highlights the opportunity to increase women’s participation in sanitation decision making, particularly in urban contexts. Women’s involvement in such decisions were widely acceptable across settlement types. As more women get involved in decisions to build toilets, highlighting this norm may encourage gender-equitable engagement in sanitation-related decisions in low-resource settings.

## Supporting information

S1 File (DOCX)

## Abbreviations

FGD: Focus group discussions
SBM: Swachh Bharat Mission
SC: Scheduled Caste
SES: Socioeconomic status
SNT: Social Norms Theory
WASH: Water, sanitation, and hygiene
