# Supplementary material for: Social beliefs and women’s role in sanitation decision making in Bihar, India: An exploratory mixed method study
Source: PLoS One. 2022 Jan 27;17(1):e0262643. doi: 10.1371/journal.pone.0262643 (PMC8794139; doi:10.1371/journal.pone.0262643)
Supplement: S1 File — (DOCX) [file pone.0262643.s001.docx]

**BIHAR Focus Group Discussion Guide**

**INTRODUCTION**

My name is _________ I have come from Kantar Public, Delhi on behalf of University of Pennsylvania. Thank you for taking the time to participate in this group discussion. We are here to do research and understand you and your community better. We want you to think about yourself, your neighbors, and the community you live in when we discuss our topics today. We will ask about sanitation, your views, your experiences and what you know to be the experience in your community.

Joining me today are (Name of Researcher) ___________ and (Name of Note-taker) ____________________. This discussion can take up to 1.30 hours. Please feel free to take a restroom break in the middle if needed. The bathroom is located ___________________.

As mentioned, we want to learn about your thoughts and experiences and also those of your community to the extent possible. There are no right or wrong answers. We want to hear different experiences, so feel free to contribute anything you think is relevant to our discussion that will help us understand your community better. If you find that you disagree with what someone is saying, we welcome that but request that you wait until they are finished talking and be respectful of them and their experience.

With your permission, for research purposes only I will audio record our conversation and our team member will take notes. Only the research team will have access to these notes and recording, and anything you say will be kept confidential. We will not ask about any personal information, your name or address. Any other identifiable detail that comes up will be removed from the documents. We also request you to not share what we discuss here today so that participants can discuss their thoughts freely.

Your participation is voluntary, and you can leave the discussion if you feel uncomfortable or need to. We can also pause the recording if you want to say something without being recorded.

Do you [everyone] consent to participate in this focus group discussion? [_____] Yes [________] No

- Total participants:

------------------------------------------------------------------------------------------------------------------------------------------------------------

| TOTAL TIME: Mins. Start time: End time: | | Notes |
| --- | --- | --- |
| SECTION A: RAPPORT BUILDING | |  |
| BACKGROUND OF THE RESPONDENT  PROJECTIVE :  COMMUNITY LAYOUT | ***Thank you for taking out time to speak with us today. I’d like to start by knowing a little more about all of you*.**  Can you please introduce yourselves with your age, years of education? 🡪 Notetaker completes survey based on responses  ***Probe:***  *Since how long have you been living in this village?*  *Who all are there in your family?*  **There is always a lot of work in the HH, how do you manage?**  ***Probe :***  *Work distribution in the HH*  *Do women undertake chores and work outside the HH?*  *Specific instances where women leave the house accompanied or may go by themselves (unaccompanied)*  **Great. I would like to know a little bit about this place**   - Can you tell me a little bit about your village/town/community?   ***Probe :***  *Population*  *Prevalent Castes along with Segregation if present*  *Family type*  *Occupation*  **MAKE A MAP OF THE LOCALITY WITH RESPONDENTS HIGHLIGHTING FIELDS, COMMUNITY TOILETS, WATER BODIES ,OD SITES, ETC.** |  |
| SECTION B: PREVALENCE OF TOILETS | |  |
| ALTERNATIVES AVAILABLE  OD  INCIDENCE  REASONS FOR OD  DESIGNATED AREAS FOR OD  OD : TIMING AND REASONS  WOMEN STEPPING OUT FOR OD  PERCIEVED BENEFITS AND CHALLENGES IN OD  OPINIONS ON OD  TOILET ACCESS VS USAGE  COMMUNITY PERCEPTIONS OF TOILET USERS  TOILET USAGE AMONG WOMEN  REASONS FOR CONTRUCTING TOILET IN THE HH  PERCIEVED BENEFITS  CHALLENGES DIFFICULTIES OF TOILET AT HOME  CHALLENGES  COMMUNITY PERCEPTION OF WOMEN USING CT’s  BARRIERS TO TOILET USAGE FOR WOMEN  DECESION MAKING AT HH  AUTONOMY FOR DECESION MAKING  FINANCIAL DECISION MAKING | Link to the above & ask the following:-   - Where do most people in this community go for defecation?   ***Probe:***  *Are toilets present in all areas of the village?*  *Personal or community ones?*  *Are they being used by everyone?*  ***Bucket responses based on :-***  *Gender—Caste—Class-- Religion.*   - Everyone has their own habits & preferences….Probe differences across gender, age groups - Speaking of women of this village, where do they go to defecate? When do they usually go?   ***Probe :***  *Any difference in sanitation behaviour basis : Age, Caste, Class, Religion*  ***Segregation by Age, Caste, Class, Religion***   - I would like to know more about the reasons for women going for open defecation.   ***Probe:***  *Habit*  *Factors related to health, purity*  *Age-old tradition, peer pressure,*  *Religious sanctity issues*  *Issue with Toilets - Small size, issue of non-availability of water, use of toilets for other purposes, cleanliness*  *No toilets in the HH*   - As you mentioned above, there seemed to be areas in the village which are specific for defecation. Which are those areas & why is this case?   ***Probe :*** *-*  *Isolated spots*  *Vacant land/ has always been used for defecation- why so?*  *Appropriate for relieving oneself- reasons.*   - Is there any usual timing? If yes, what? What are the reasons for the same?   ***Probe:***  *Do they go alone or are accompanied by someone?*  *Based on different age-groups & timings (corresponding).*  *Socio-cultural norms & social diktats.*  Link to the question asked in section 1 and ask:   - Are there instances when women are accompanied and not accompanied by anyone? Which are these?   ***Probe:***  *Comfort levels in going accompanied/unaccompanied*  *Reasons for going accompanied / unaccompanied.*   - Any challenges faced in OD– seasonal, aesthetics, cultural? - Is the location for OD selected or is it imposed? - Does distance play a role in it? How?   ***Probe:***  *Location of OD, Cultural factors (marriage, prevalence of Purdah system, safety issues)*   - Are there any perceived benefits of open defecation? - What is the opinion of community at large regarding open defecation? Do you feel other people disapprove of openly defecating?   ***Probe:***  *Opinions across Genders, Age groups*   - In what ways do they express their opinion?   ***Probe :***  *Disapproval shown via gestures, words etc.*     - Can you share any specific example where you think someone has shown their opinion of OD? Who would disapprove & of whom?   ***Probe*** *:*  *Caste, Gender or religious specific nuances*   - Suppose people here (***probe for both people in the HH and outside the HH)*** are openly disapproving of you/someone who defecates in the open. Do you think it would make you /the said person change their behaviour? Why/Why not?   ***Probe :***   - *Caste, Gender or religion specific nuances* - *Who in your village or elsewhere has the ability to make people change their behaviour? (authority figures –without a formal designation , neighbors , elders , other aspirational figures)* - We have seen in many villages that there are sometimes people who have access to toilets but they don’t use them. Are there any such cases in this community too? What could be the reasons?   ***Probe:***  *Peer Pressure*  *Habit, cultural aspects like new bride at home*  *Condition of toilet, non-availability of water, convenience.*   - Do you think other people disapprove of men using a household toilet? Why/Why not? - If they see or know a man who uses a household toilet what will they say or/and do?   ***Probe :***  *Say anything? Do anything?*  *Ask for specific examples-- who will disapprove and of whom?*   - Are there women who use toilets? Majorly do they use:- - *Indoor toilets/ located inside houses* - *Outdoor toilets/ located outside houses* - *Community/ Public toilets*   **NOTE: QUESTIONS BELOW TO BE ASKED ONLY TO RESPONDENTS WITH TOILETS IN THEIR HH’S**  ***I would now ask about those who use their private toilets:-***   - What were the reasons for building a toilet in your household?   ***Probe****:*  *Privacy, Status symbol, Aspirations*   - Who decided to get it constructed?   ***Probe:***  *Were you or any other female from your family, part of decision making process?*  *Who was the key - influencer? Whose idea is it?*  *Who was the key decision maker? Chief wage earner, Patriarch, Head of the HH*  *Who finally ensures /works towards building the toilet?*   - What are the benefits of having a toilet at home?   ***Probe:***  *Perceived difference b/w toilet vs. OD*  *Felt change in life since using toilets?*  Suppose you have a toilet at home.   - Who would be responsible for its upkeep? - Are there challenges/difficulties you anticipate would come with it? Why/Why not? If yes, what would they be?   ***Probe****:*  *Toilet inside the HH and outside the HH*  *Maintaining the toilet- water, hygiene etc.*  *Cultural issues with women using same toilet as men*  **MODERATOR TO ASK THIS SECTION, ONLY IF COMMUNITY TOILETS EXIST IN THE LOCALITY.**   - Approximately, how many women use community toilets?   ***Probe:***  *Women of which age group?*  *When do they generally use them?*  *Please tell more about these toilets (cleanliness, Hygiene etc.)*   - What are the challenges of using a community toilet?   ***Probe:***  *Permission*  *Going unaccompanied, fear, safety issues, other factors?*   - What is the opinion of community at large about women using community toilets? - If someone sees a women going to use community toilets, how would he/ she react?   ***Probe:***  *Incidences of disapproval*  *Who all disapprove*  *Ways of disapproval : Gestures, words, deeds*  *Reasons of disapproval*   - In this village, do people have any reservations with women going unaccompanied for defecation? - How do they react? Any examples? - What do you think are probable reasons?   ***Probe:***  *Any differences by religion, caste & class.*   - Suppose people here (***probe for both people in the HH and outside the HH)*** are openly disapproving of you or a woman using a community toilet. Do you think it would make you/ the said person change his behaviour? Why/Why not?   ***Probe :***   - *Caste, Gender or religion specific nuances* - *Who in your village or elsewhere has the ability to make people change their behaviour? (authority figures –without a formal designation , neighbors , elders , other aspirational figures)* - Suppose a woman wants to use a toilet. What are the barriers that she might face that prevents access to toilet?   ***Probe:***  *Lack of resources*  *Lack of say in household decisions*  *Space constraints/ other issues*  *Cultural factors*     - In this village, generally who take financial decisions in a household? Probe for outliers. - What kind of financial decisions do men of village generally take? Can you share experience from your household? - Are there any financial decisions that women take? Please share experience of your household. - Suppose someone wants to get a toilet constructed. Who in the household, would decide to spend money on that toilet?   ***Probe:***  *What process would that household follow to get it constructed?*  *Whom would they contact to get it constructed?*  *Who will decide to pay money?*  *How will it get built?*   - Do you think women can take the decision to get a toilet constructed independently? Why/ why not?     ***Probe:***  *If yes, what are the facilitating factors – support, resources, education, awareness, etc.*  *If not, who are they required to take permission from?*  *If a woman tries to take such decisions independently, what is the reaction of other members of the family? Relatives & larger village community?*   - Generally, what is the attitude of community towards women taking larger financial decisions like getting a toilet constructed? What are the reasons? - Suppose if a woman independently gets a toilet built for her household without consulting her husband & other family members, how would members of the family react to it? What would they do or say?   ***Probe:***  *Reaction of community at large.*  *Any specific people who would approve or disapprove.*  *Reasons for such reactions.*   - Suppose people here (***probe for both people in the HH and outside the HH)*** are openly disapproving of you/someone who is pushing to get a toilet. Do you think it would make you/the said person change her behaviour? Why/Why not? - Who do you think can support her? How and why? (check for local NGOs , FLWs , SHGs etc)   ***Probe :***   - *Caste or religion specific nuances* - *Who in your village or elsewhere has the ability to convince the people opposing you/the woman? (Authority figures –without a formal designation , neighbors , elders , other aspirational figures)* - If a woman has money or if she receives a good sum of money (>10,000 INR) from some source:   ***Probe:***  *On what would the money be spent?*  **ASK RESPONDENTS TO PRIORITIZE THEIR SPEND IN THREE BUCKETS, ASK THEM WHERE THEY WILL BE ABLE TO SPEND WITH AND WITHOUT PERMISSION**  *Will the construction of individual toilet be a priority?* |  |

***Conclusion***

Thank you for your thoughts in this discussion. Does anyone have anything else that you would like to share?

Yes: _______________________________________________________ [___] No

This concludes our discussion today. Please remember our conversation here today is confidential and it will not be shared publicly in a way that identifies you or this community.

***Do you have any questions?***

**THANK YOU**
